# Posttraumatic growth and well-being among people living with HIV: A systematic review and meta-analysis in recognition of 40 years of HIV/AIDS

**DOI:** 10.1007/s11136-021-02990-3

**Published:** 2021-09-14

**Authors:** Małgorzata Pięta, Marcin Rzeszutek

**Affiliations:** Faculty of Psychology, University of Warsaw, Stawki 5/7, 00-183 Warsaw, Poland

**Keywords:** Posttraumatic growth, Well-being, Distress, HIV/AIDS, Adults, Systematic review, Meta-analysis

## Abstract

**Objectives:**

This systematic review and meta-analysis aimed to synthesize, analyze, and critically review existing studies on the relationship between posttraumatic growth (PTG) and psychological well-being (operationalized either via positive or negative well-being indicators) among people living with HIV (PLWH). We also investigated whether this association varies as a function of socio-demographic, clinical characteristics, and study publication year.

**Method:**

We conducted a structured literature search on Web of Science, Scopus, MedLine, PsyARTICLES, ProQuest, and Google Scholar. The most important inclusion criteria encompassed quantitative and peer-reviewed articles published in English.

**Results:**

After selection, we accepted 27 articles for further analysis (*N* = 6333 participants). Eight studies used positive indicators of well-being. The other 19 studies focused on negative indicators of well-being. Meta-analysis revealed that there was a negative weak-size association between PTG and negative well-being indicators (*r* = − 0.18, 95% CI [− 0.23; − 0.11]) and a positive medium-size association between PTG and positive well-being measures (*r* = 0.35, 95% CI [0.21; 0.47]). We detected no moderators.

**Conclusions:**

The present meta-analysis and systematic review revealed expected negative and positive associations between PTG and negative versus positive well-being indicators among PLWH. Specifically, the relationship between PTG and positive well-being indicators was more substantial than the link between PTG and negative well-being measures in these patients. Finally, observed high heterogeneity between studies and several measurement problems call for significant modification and improvement of PTG research among PLWH.

Roughly a quarter century has passed since Tedeschi and Callhoun [1] started to empirically examine the famous philosophical thought by Nietzsche that *what doesn’t kill you makes you stronger*, which resulted in a new line of research on posttraumatic growth (PTG). Over these years, several theoretical models of PTG emerged (e.g., [1–6] and literarily hundreds of studies on growth in versatile trauma survivors have been conducted (see reviews and meta-analyses by [7–9]. Despite all these empirical strain, numerous theoretical and methodological challenges in PTG research still preclude us from answering many fundamental questions [10]. One of them is how perceived growth among people exposed to traumatic events translates into their psychological functioning [11, 12]. Despite intuitively apparent assumptions on the adaptive role of PTG, studies have documented the positive, negative, and null associations between PTG and well-being after trauma (see reviews and meta-analyses by [3, 9, 11]). Zoellner and Maercker [12, p. 631] best summarize the significance of this latter problem: “If posttraumatic growth is a phenomenon worthy to be studied in clinical research, it is assumed to make a difference in people’s lives by affecting levels of distress, well-being, or other areas of mental health. If it does not have any impact, then, PTG might just be an interesting phenomenon possibly belonging to the areas of social, cognitive, or personality psychology.” Therefore, our systematic review and meta-analysis is intended to fill this research gap by analyzing the PTG-well-being association in the clinical sample of people living with HIV (PLWH).

The issues mentioned above are of particular interest within much understudied and controversial line of PTG research, i.e., PTG in case of life-threatening illness [13, 14]. The fourth edition of the Diagnostic and Statistical Manual of Mental Disorders (DSM-IV; [15]) classifies the diagnosis of a somatic disease and struggling with its consequences as a traumatic event and a trigger of posttraumatic stress disorder (PTSD; e.g., [16–18]). Nevertheless, from its beginning, the topic mentioned above posed huge controversies related to the problems in fulfilling traumatic stressor criteria by people coping with a terminal illness, as well as the lack of precise mechanisms linking PTSD and illness-related trauma (e.g., [19–21]. It is controversial especially in the light of the most recent PTSD criteria in DSM-5 [22]. A few years ago, Edmondson [23] formulated the Enduring Somatic Threat model of PTSD, which was the first theoretical model of PTSD in the context of life-threatening illness. According to this model, traumatic symptoms observed among patients struggling with such illness are a complex, continuous process, which several time perspectives can describe: the past (e.g., diagnosis), present (e.g., painful treatment, full of side effects, stigmatization), and future (e.g., awareness of constant life threat).

The posttraumatic experiences are particularly prevalent among PLWH (see reviews and meta-analyses by [24–26]). Psychological distress reported by people with HIV has a complex and multifactorial nature. The necessity of life-long adherence to treatment regimes, the unpredictability of the course of HIV infection, the persistently strong social stigma directed towards PLWH and sometimes also with pre-morbid trauma history are chronic stressors that are related to various psychopathological symptoms among this group of patients, including PTSD [25, 27–29]. Finally, we should emphasize that the long-lasting HIV-related distress associated with the experience of this disease may negatively affect the course of HIV infection, including a decline in immunological functioning, which increases the risk of AIDS [30].

However, along with the tremendous progress in the treatment of HIV/AIDS and its transformation from a terminal to a chronic and manageable medical problem [31, 32], more researchers have started to study the positive side of trauma that accompanies HIV-related PTG [14, 26]. However, according to a recent review by Rzeszutek and Gruszczyńska [33], these studies present a very incomplete, thematically heterogonous, and inconsistent picture of growth predictors among PLWH. One of the most critical research gaps is whether and how PTG translates into PLWH’s psychological functioning, including an individual’s psychological well-being, operationalized via positive indicators (e.g., quality of life, life satisfaction, etc.) considering negative measures (depression, distress, etc.), by controlling the socio-demographic and clinical covariates. Sawyer et al. [14] conducted the last and only meta-analysis on this topic more than a decade ago when research on PTG among PLWH was really in its infancy. Thus, its final remarks need to be updated.

## Objective

The general aim of this systematic review and meta-analysis was to synthesize, analyze, and critically review existing studies on the relationship between posttraumatic growth and psychological well-being among PLWH. We focused on the vast operationalization of the well-being concept positively, including quality of life and mainly health-related quality of life, satisfaction with life, and affective well-being. Regarding negative HIV-related well-being aspects, we concentrated on symptoms of depression, anxiety, posttraumatic stress disorder, and experienced HIV-related stigma.

In the meta-analytic part, we examined the overall strength and direction of associations between PTG and well-being, also looking for their possible moderators like a study’s year of publication, socio-demographic data, and HIV-related clinical variables. This latter clinical variable is particularly in the center of our interest, as it occurred to be the strongest moderator of the association between PTG and both positive and negative aspects’ adjustment to HIV infection [14]. We found the same trend pointing to the time elapsed since traumatic event as a PTG well-being moderator in meta-analytic reviews across a wide range of traumatic events [7].

## Method

### Systematic review and meta-analysis protocol

**Fig. 1:**
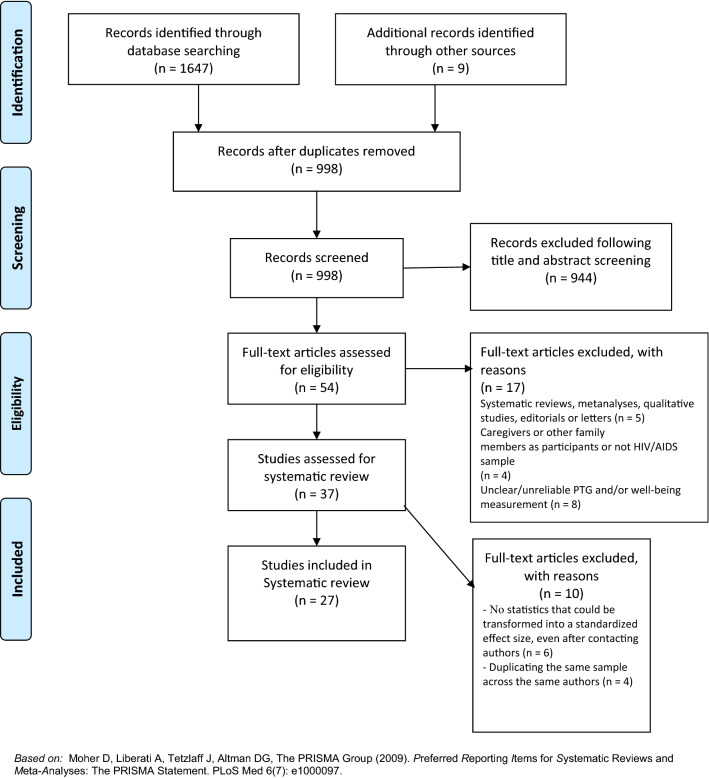
PRISMA flow diagram Moher et al. [34]

We performed the literature search and review based on the standards of the Preferred Reporting Items for Systematic Reviews and Meta-analyses (PRISMA) statement ([34]; see Fig. 1). We searched the following databases on 06 March 2021: Web of Science, Scopus, MedLine, PsyARTICLES, and ProQuest. We also used Google Scholar as an additional source of *grey literature* [35]. In Boolean algebra, the query consisted of the following terms: (*hiv OR *(*acquired AND immunodeficiency AND syndrome*)* OR *(*human AND immunodeficiency AND virus*)* OR *(*hiv/aids*)* AND *(*ptg OR *(*posttraumatic AND growth*)* OR *(*posttraumatic AND growth*)* OR *(*benefit AND finding*)* OR thriving*)* AND *(*well-being OR well-being OR *(*well AND being*)* OR *(life AND satisf*) OR life-satisf* OR (*quality AND life*)* OR *(*life AND quality*)* OR life-quality*) OR depress* OR anxi* OR (*posttraumatic AND stress*)* OR *(*posttraumatic AND stress*)* OR ptsd*). We limited the search to papers written in English.

### Study selection criteria

Apart from the English-language criterion, the studies must meet the following requirements to be included in the systematic review and subsequently in the meta-analysis:

(1) Type of study—We accepted only peer-reviewed, quantitative, empirical articles that measured the relationship between PTG and psychological functioning among PLWH. We excluded other systematic reviews and meta-analyses, as well as editorials, letters, and qualitative studies.

(2) Participants—We included studies with adult HIV/AIDS patients, with no restriction on gender, sexual orientation, ethnicity, or stage of the disease and its duration. We also included studies where participants were composed of PLWH and patients with other chronic diseases. We excluded studies that described caregivers of PLWH or their family members.

(3) Methodology—We included only studies with psychometrically sound measurements of PTG and well-being or distress outcomes. We reported any of the following statistics: correlation coefficients, sample sizes, regression coefficients, or other statistics that could become a standardized effect size. We excluded studies with no psychometric PTG and well-being or distress measures (i.e., with ad hoc self-created measures by the authors) and studies without sufficient statistics for performing a meta-analysis, even after contacting the authors.

(4) Quality of study—We followed the Quality Assessment Tool for Observational Cohort and Cross-Sectional Studies [36], composed of 14 criteria and particular questions regarding meeting these criteria. The potential answers are Yes, No, Cannot Determine, Not applicable, and Not Reported. A score of > 11 is a sign of good quality, 7–10 fair quality, and < 7 poor quality. Two independent evaluators examined the studies (see Results and Fig. 1). The evaluators were particularly looking for validated measures with psychometric data, clear definition or operationalization of PTG and well-being or distress outcomes, controlled for a sufficient amount of socio-demographic and HIV-related covariates, and appropriate statistics to calculate effect sizes.

### Data extraction and statistical analysis

We conducted the meta-analysis with library “meta” in the R Statistics 4.03 software environment [37]. We calculated the Pearson correlation coefficients as effect size measures. We transformed the unstandardized and standardized regression coefficients from single studies to Pearson correlation coefficients according to the recommendations of Lipsey and Wilson [38] using library “esc.” We performed outlier diagnostics on Baujat plot [39]. We also performed the Graphic Display of Heterogeneity (GOSH) plot [40] analysis. We calculated publication bias with a funnel plot and investigated the potential moderators in meta-regression [41].

## Results

### Screening and eligibility

Initially, we reached 1656 titles and abstracts via electronic databases search, including 671 hits on Web of Science, 414 hits on MedLine, 324 Scopus, 223 hits on Proquest, 15 hits on PsyARTICLES, and nine hits on Google Scholar. After removing duplicates, we reached 998 potentially eligible articles for further screening. After careful title and abstract screening by two independent reviewers, 54 full articles remained for the assessment. Using the exclusion criteria, we eliminated 17 papers. We excluded ten studies because they did not have statistics for meta-analysis even after contacting their authors or duplicated samples present in other studies. In the end, we accepted 27 articles for systematic review and meta-analysis.

We managed to find articles dating from 2004 to 2021. The total sample size was *n* = 6333 PLWH, including 4358 men, 1962 women, and 13 participants, who declared the “other” option in this aspect. Finally, 81% (22/27) of the analysed studies were cross-sectional.

### Measures

The most common measure of growth after trauma was the Posttraumatic Growth Inventory (PTGI; [1], sometimes in the short version). Much less utilized were the Benefit Finding Scale [42, 43], Stress-Related Growth Scale (SRGS,[44], Flourishing Scale [45], and Silver Lining Questionnaire (SLQ; [46]). Regarding well-being outcomes, the authors focus predominantly on health-related quality of life, life satisfaction, self-esteem, or affective well-being assessed by, e.g., The 20-Item Short-Form Health Survey (SF-20; [47], Satisfaction with Life Scale (SWLS,[48], Self-esteem (Rosenberg Self-Esteem Scale,[49], and Positive and Negative Affect Schedule (PANAS,[50]. Finally, HIV-related distress issues were operationalized most often by depression (e.g., Center for Epidemiological Studies Depression Scale or CES-D,[51], HIV/AIDS stigma (e.g., HIV-Stigma Scale,[52], and HIV-related PTSD (e.g., PTSD Factorial Version or PTSD-F,[53].

At this point, it is vital to highlight a few remarks on HIV-related PTG, well-being, and distress operationalization. First, the vast majority of studies (even if they did not use PTGI explicitly) followed the PTG model by Tedeschi and Calhoun [1, 6]. In this model, PTG is both an outcome of dealing with trauma or a process of coping with a traumatic event, which may eventually lead to positive or negative changes in the long-term well-being of a trauma survivor. Thus, on the one hand, 67% of eligible studies (18/27) applied this first mode of PTG operationalization, i.e., PTG was the outcome variable, and the well-being or distress variables were its predictors. On the other hand, 33% of the studies (8/27) deviated and treated PTG as a predictor of well-being and distress. Second, in the eligible studies, the authors performed statistical analysis on the global PTG score. The relevant statistical details for the meta-analysis were available only for this global score, despite assessing PTG subscales. We observed the same attitude in the HIV-related well-being and distress issues we studied.

Table 1 summarizes all the details related to the systematic review of our 27 final studies. The studies included in the review encompassed both positive and negative indicators of HIV-related well-being. We conducted meta-analysis separately on the studies based on either positive versus negative well-being indicators in this patient group.

**Table 1:** Summary of the relationship between posttraumatic growth and psychological well-being among PLWH in the final 27 eligible studies

Author	Year	Study design	PTG conceptualization and measure	PTG as outcome vs. predictor variable	Well-being/distress conceptualization and measure	Sample (gender)	Sample (mean age in years)	Sexual orientation	Ethnicity	Soc-dem variables	HIV-related clinical variables	Significant sociomedical covariates
Milam et al [54]	2004	Longitudinal	Posttraumatic Growth: Posttraumatic Growth Inventory (PTGI; [1]-short, 11-items)	outcome	Depression (Center for Epidemiological Studies Depression scale; CES-D; [51]	434 (M = 355; F = 79; O = 0)	38,35	HT = 113 HM = 269 BI = 52	39.5% C 36.8% 17% AA 6.7% O	51.7% HE 40.6% SR 38.3% EM	CD4 mean: 273,16; 52% detectable viral load; 6.39 years since diagnosis/ treatment years; 46% AIDS	No significant covariates
Milam et al [55]	2006	Longitudinal	Posttraumatic Growth: Posttraumatic Growth Inventory (PTGI; [1]-short, 11-items)	outcome	Depression; Center for Epidemiological Studies Depression scale (CES-D; [51]	412 (M = 363; F = 49; O = 0)	39	HT = 94 HM = 276 BI = 42	38.8% C 40.3% H 14.8% AA 6.1% O	24.3% EM	CD4 mean: 472,8; 42% detectable viral load; 6.4 years since diagnosis/treatment years	*Gender*: higher PTG levels in women than in men; *ethnicity*: highest PTG levels in Hispanics and positive association between PTG and *CD4 levels* in this group; negative association between PTG and *detectable viral load*
Carrico et al [56]	2006	Cross-sectional	Benefit finding; The Benefit Finding Scale for breast cancer [42]	predictor	Depression; Beck Depression Inventory (BDI)	264 (F = 134; M = 130; O = 0)	40	n/a	25% C 49% AA 13% H	47% HE 34% EM	CD4 mean: 453,1; 42% detectable viral load; 7.7 years since diagnosis/ treatment years	No significant covariates
Łuszczyńska et al [57]	2007	Cross-sectional	Benefit finding: The Benefit Finding Scale for breast cancer [42]	predictor	Quality of life; The 20-Item Short-Form Health Survey (SF-20; [47]	104 (F = 66; M = 38; O = 0)	34,73	HT = 104	100% Indian	n/a	5.1 years since diagnosis/ treatment years	Positive association between PTG levels and participants’ *age* and *years of treatment*
Siegel, & Schrimshaw [58]	2007	Cross-sectional	Benefit finding: Psychological Thriving Scale (Abraido-Lanza et al., 1998)	predictor	Depression; Mental Health Inventory (MHI)	138 (F = 138 M = 0 O = 0)	37,6	n/a	28% C 38% AA 34% Puerto Rican	37% HE 17% SR 21% EM	CD4 mean: 327,4; 36% detectable viral load; 7.3 years since diagnosis/ treatment years; 51% AIDS status	No significant covariates
Littlewood et al [59]	2008	Cross-sectional	Benefit finding; The Benefit Finding Scale for breast cancer [42]	predictor	Depression; Center for Epidemiological Studies Depression Scale (CES-D; [51]	221 (F = 97; M = 124; O = 0)	40	n/a	46% C 42% AA 4% NA 4%AP 4% O	27% HE 33% EM	CD4 mean: 500,1; 22% detectable viral load; 7 years since diagnosis/ treatment years; 61% AIDS status	*Gender, ethnicity, AIDS status*: higher PTG levels and lower depression levels in women, African Americans, and AIDS stage patients
Kraaij et al [60]	2008	Cross-sectional	Personal Growth; Personal Growth Scale (PGS; Garnefski et al., 2008)	outcome	Positive reappraisal (Cognitive Emotion Regulation Questionnaire (CERQ)	104 (F = 0; M = 104; O = 0)	50,1	HT = 0; HM = 96 BI = 8	100% C	50% HE 50% SR 50% EM	10.1 years since diagnosis/ treatment years	No significant covariates
Cieślak et al [61]	2009	Cross-sectional	Posttraumatic Growth; Posttraumatic Growth Inventory (PTGI; [1])	outcome	PTSD (PTSD checklist; PCL-S)	90 (F = 30; M = 57; O = 3)	41,61	n/a	n/a	66.7% HE 17.2% SR 25.5% EM	9.71 years since diagnosis/ treatment years	No significant covariates
Murphy & Hevey [62]	2013	Cross-sectional	Posttraumatic Growth; Posttraumatic Growth Inventory (PTGI; [1])	outcome	HIV/AIDS stigma (The internalized AIDS-related stigma scale; IA-RSS)	74 (F = 18; M = 56; O = 0)	39,79	HT = 28 HM = 40 BI = 6	87% C 13% AA	86.3% HE 56.9% SE 72.6% EM	7.89 years since diagnosis/ treatment years	No significant covariates
Kamen et al [63]	2016	Cross-sectional	Posttraumatic Growth; Posttraumatic Growth Inventory (PTGI; [1])	outcome	HIV/AIDS stigma (HIV-Stigma Scale; [52]	334 (F = 87; M = 247; O = 0)	45,9	HT = 130 HM = 204 BI = 0	32.6% C 38.6% AA 16.8% H 5.7% Asian 6.3% O	32.9% HE 14.4% EM	n/a	*Gender:* higher PTG levels in women than in men; *ethnicity:* highest PTG levels in African Americans, lowest in Caucasians; *sexual orientation:* highest stigma levels in heterosexuals
Lyons et al [64]	2016	Cross-sectional	Flourishing; Flourishing Scale [45]	outcome	HIV/AIDS stigma (The internalized AIDS-related stigma scale; IA-RSS)	357 (F = 0; M = 357; O = 0)	30,4	HT = 0 HM = 357 BI = 0	100% C	21.4% HE 47.1% SR 53% EM	11.1 years since diagnosis/ treatment years	*Employment:* lower flourishing level and higher stigma in unemployed participants
Rzeszutek et al [27]	2016	Cross-sectional	Posttraumatic Growth; Posttraumatic Growth Inventory (PTGI; [1])	outcome	PTSD; PTSD Factorial Version (PTSD-F; [53]	250 (F = 44; M = 206; O = 0)	39,35	n/a	100% C	n/a	7.29 years since diagnosis/ treatment years	*Gender:* higher PTG and PTSD levels in women; gender moderated association between PTG and PTSD
Zeligman et al [65]	2016	Cross-sectional	Posttraumatic Growth; Posttraumatic Growth Inventory (PTGI; [1])	outcome	HIV/AIDS stigma (HIV-Stigma Scale; [52]	126 (F = 39; M = 84; O = 3)	49,1	n/a	44% C 44% AA 6% H 2% AP 4% O	61% HE	10.1 years since diagnosis/ treatment years	*Education:* negative association between education level and PTG
Fekete et al [66]	2016	Cross-sectional	Benefit Finding; Benefit Finding Scale (BFS; [43])	predictor	Depression; Center for Epidemiological Studies Depression Scale (CES-D; [51]	167 (F = 42; M = 121; O = 4)	43,6	HT = 77 HM = 73 BI = 17	52% C 48% AA	23.4% HE 37.7% SR 47.3% EM	11.81 years since diagnosis/ treatment years	*Ethnicity:* negative association between benefit finding and depression only in Caucasians
Garrido-Hernansaiz et al [67]	2017	Longitudinal	Posttraumatic Growth; Posttraumatic Growth Inventory (PTGI; [1]; short version)	outcome	HIV/AIDS stigma (The HIV Internalized Stigma Scale; [67])	119 (F = 12; M = 116; O = 1)	32,73	HT = 2 HM = 104 BI = 13	4% C 96% H	69% HE 13% SR 75% EM	1 year since diagnosis/ treatment year	No significant covariates
Rzeszutek [68]	2017	Longitudinal	Posttraumatic Growth; Posttraumatic Growth Inventory (PTGI; [1])	outcome	Affective well-being (Positive/negative affect; PANAS-X, [50]	82 (F = 12; M = 70; O = 0)	40,5	n/a	100% C	62.3% HE 59.8% SR 64.6% EM	CD4 mean: 645,73, 7.39 years since diagnosis/ treatment years, 19.50% AIDS status	*AIDS status* associated with lower PTG level, lower positive affect level and higher negative affect level; *employment* associated negatively with negative affect
Rzeszutek et al [69]	2017	Longitudinal	PTSD (PTSD Factorial Version; PTSD-F; [53]	outcome	PTSD (PTSD Factorial Version; PTSD-F; [53]	73 (F = 5; M = 68; O = 0)	38,77	n/a	100% C	n/a	6.42 years since diagnosis/ treatment years	No significant covariates
Chang et al [70]	2018	Cross-sectional	Depression; Center for Epidemiological Studies Depression Scale (CES-D; [51]	predictor	Stress-related growth; Posttraumatic Growth Inventory (PTGI; [1])	178 (F = 81; M = 97; O = 4)	34,52	n/a	100% Indian	17% HE 4.7% SR	n/a	No significant covariates
Ye et al [71]	2018	Cross-sectional	Posttraumatic Growth; Posttraumatic Growth Inventory (PTGI; [1])	outcome	PTSD (Impact of Events Scale; IES)	140 (F = 0; M = 140; O = 0)	26,6	HT = 0 HM = 140 BI = 0	100% AP	82.1% HE 1.5% SR 68.6% EM	1 year since diagnosis/ treatment year	No significant covariates
Lau et al [72]	2018	Cross-sectional	Posttraumatic Growth; Posttraumatic Growth Inventory (PTGI; [1]; short 11-items)	outcome	Negative emotional illness perception (The revised Illness Perception Questionnaire for HIV (IPQ-R-HIV)	225 (F = 0; M = 225; O = 0)	28,8	HT = 0 HM = 162 BI = 63	100% Asian	44.4% HE 33.3% SR 62.2% EM	CD4 mean: 388,3; 1 year since diagnosis/ treatment year	*Age* associated negatively with PTG level
Dibb et al [73]	2018	Cross-sectional	Posttraumatic Growth; Posttraumatic Growth Inventory (PTGI; [1])	predictor	Life satisfaction; The Satisfaction with Life Scale [48]	73 (F = 23; M = 48; O = 2)	40,26	HT = 32 HM = 31 BI = 10	64% C 21.7% AA 8.7% AP 5.6% O	24.2% SR	11.79 years since diagnosis/ treatment years	No significant covariates
Rzeszutek et al [74]	2019	Cross-sectional	Posttraumatic Growth; Posttraumatic Growth Inventory (PTGI; [1])	outcome	Life satisfaction; The Satisfaction with Life Scale [48]	470 (F = 82; M = 388; O = 0)	40,02	n/a	100% C	53.2% HE 62.8% SR 71.1% EM	593,12 CD4 mean; 7.9 years since diagnosis/ treatment years; 16.20% AIDS status	*Relationship status:* stronger association between SWLS and PTG in single participants; *education:* stronger association between SWLS and PTG in less educated participants
Onu et al [75]	2020	Cross-sectional	Posttraumatic Growth; Posttraumatic Growth Inventory-Short Form (PTGI-SF; Cann et al., 2010)	predictor	Quality of life; Patient-Reported Outcome Quality of Life-HIV (PROQOL-HIV)	201 (F = 138; M = 63; O = 0)	40,1	n/a	100% African	19.4% HE 60.7% SR 100% EM	1 year since diagnosis/ treatment year	*Years since diagnosis* associated positively with PTG level; *gender:* lower PTG level in women; *education* related negatively with PTG
Drewes et al [76]	2020	Cross-sectional	Adversial growth; Silver Lining Questionnaire (SLQ; [46]	predictor	Depression; The Patient Health Questionnaire-4 (PHQ-4)	839 (F = 101; M = 738; O = 0)	56,9	HT = 67 HM = 671 BI = 101	n/a	44.7% HE	16.7 years since diagnosis/ treatment years	No significant covariates
Ogińska-Bulik, & Kobylarczyk [77]	2020	Cross-sectional	Posttraumatic Growth; Posttraumatic Growth Inventory (PTGI; [1]	outcome	Cognitive functioning: ruminations (Event Related Rumination Inventory)	64 (F = 25; M = 39; O = 0)	34,2	n/a	100% C	n/a	5.3 years since diagnosis/ treatment years	No significant covariates
Yang et al [78]	2020	Cross-sectional	Posttraumatic Growth; Posttraumatic Growth Inventory (PTGI; [1]; short version)	outcome	Resilience; The 25-item Connor–Davidson Resilience Scale (CD-RISC)	546 (F = 546; M = 0; O = 0)	36,2	n/a	100% Asian	7.3% HE 76.9% SR	CD4 mean: 378,2; 4,6% detectable viral load; 10.2 years since diagnosis/ treatment years; 36% AIDS status	*Age* and *years since diagnosis* associated positively with PTG level
Maqsood et al [79]	2021	Cross-sectional	Stress-Related Growth; Stress-Related Growth Scale (SRGS; [44]	outcome	Self-esteem; Rosenberg Self-Esteem Scale; [49]	248 (F = 124; M = 124; O = 0)	34,2	n/a	100% Pakistanis	33,9% HE	n/a	No significant covariates

*F* female, *M* male, *O* other, *HT* heterosexual, *HM* homosexual, *BI* bisexual, *C* Caucasian, *H* Hispanic, *AA* African American, *NA* Native American, *AP* Asian/Pacific, *HE* Higher education, *SR* stable relationship, *EM* employed

## Meta-analysis: PTG and negative HIV-related well-being indicators

### Diagnosis of outliers and influencing cases

**Fig. 2:**
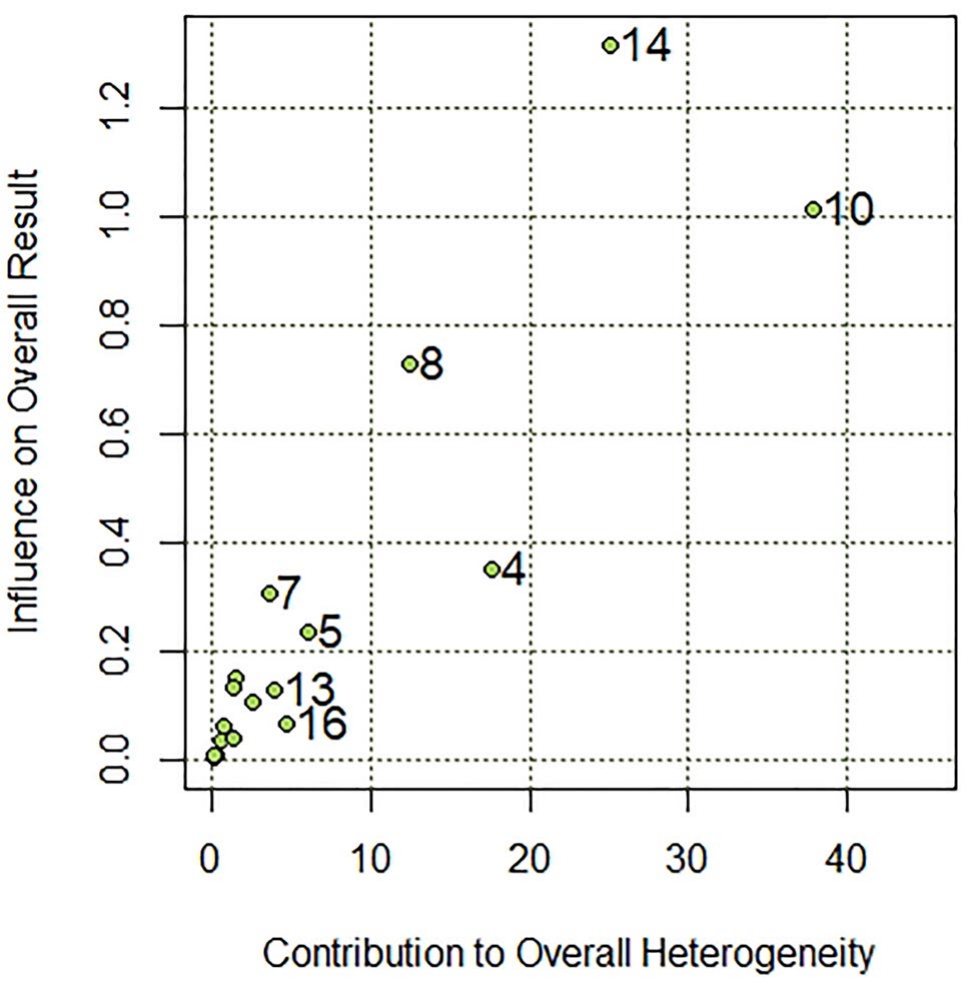
Heterogeneity diagnostics on Baujat plot

First, we conducted a meta-analysis for the studies based on negative indicators of well-being (*k* = 19). We identified outliers or studies yielding observed effects outlying or well-separated from the rest of the data with a Baujat plot. Figure 2 depicts the results of the Baujat plot. The plot shows the contribution of each study to the overall Q-test statistic for heterogeneity and the influence of each research on the overall result.

**Fig. 3:**
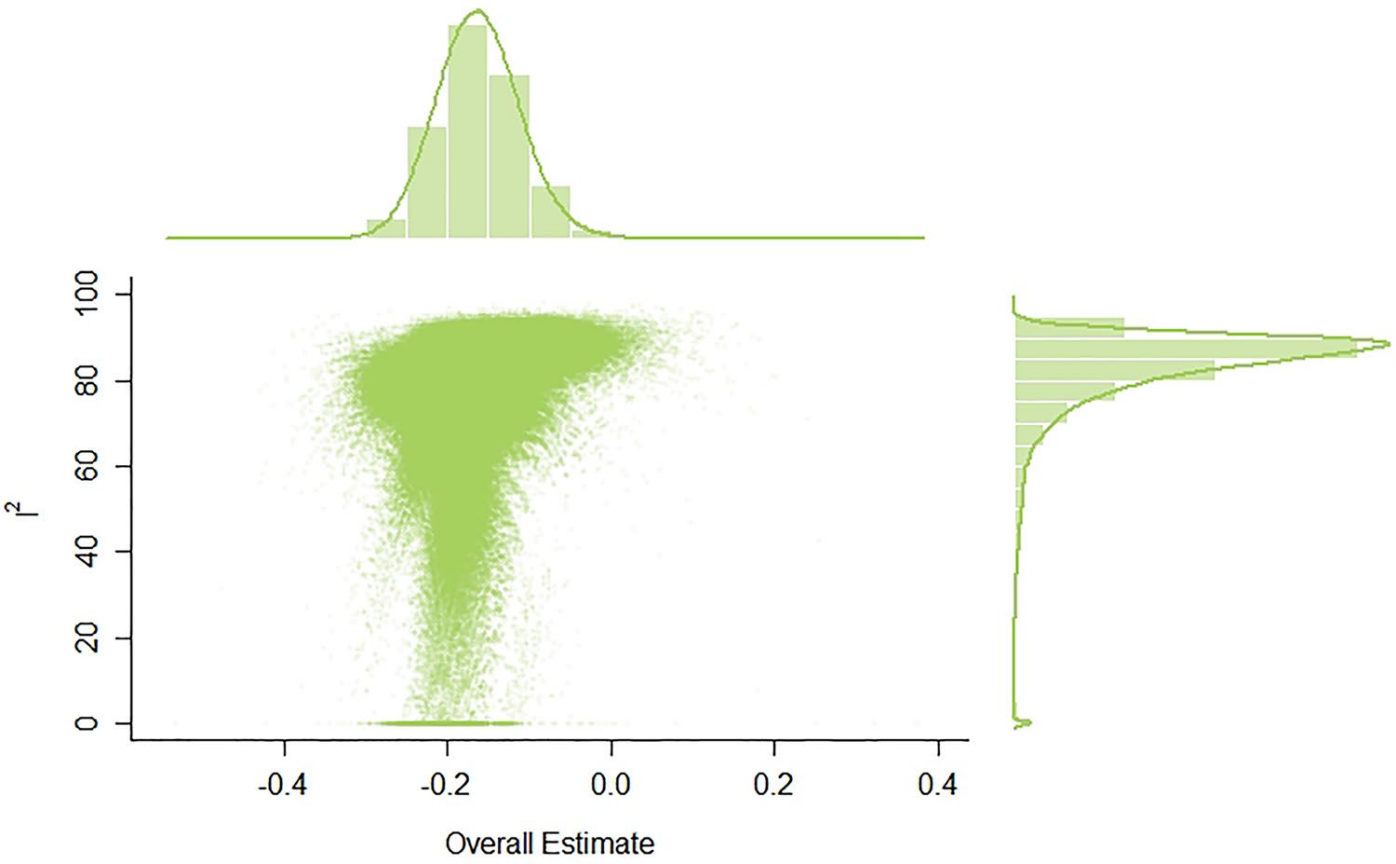
GOSH plot analysis. In order to explore patterns of heterogeneity the same meta-analysis model was fitted to all possible subsets of included studies. I^2^: I-squared statistic of heterogeneity

As can be seen, Study 13 [67] and Study 16 [72] significantly deviated from all the other studies. What is more, GOSH plot analysis aimed to detect outliers and influential studies also revealed highly skewed distribution and multimodality (see Fig. 3). For this reason, we excluded both Study 13 and Study 16 from a further meta-analysis.

### Publication bias

**Fig. 4:**
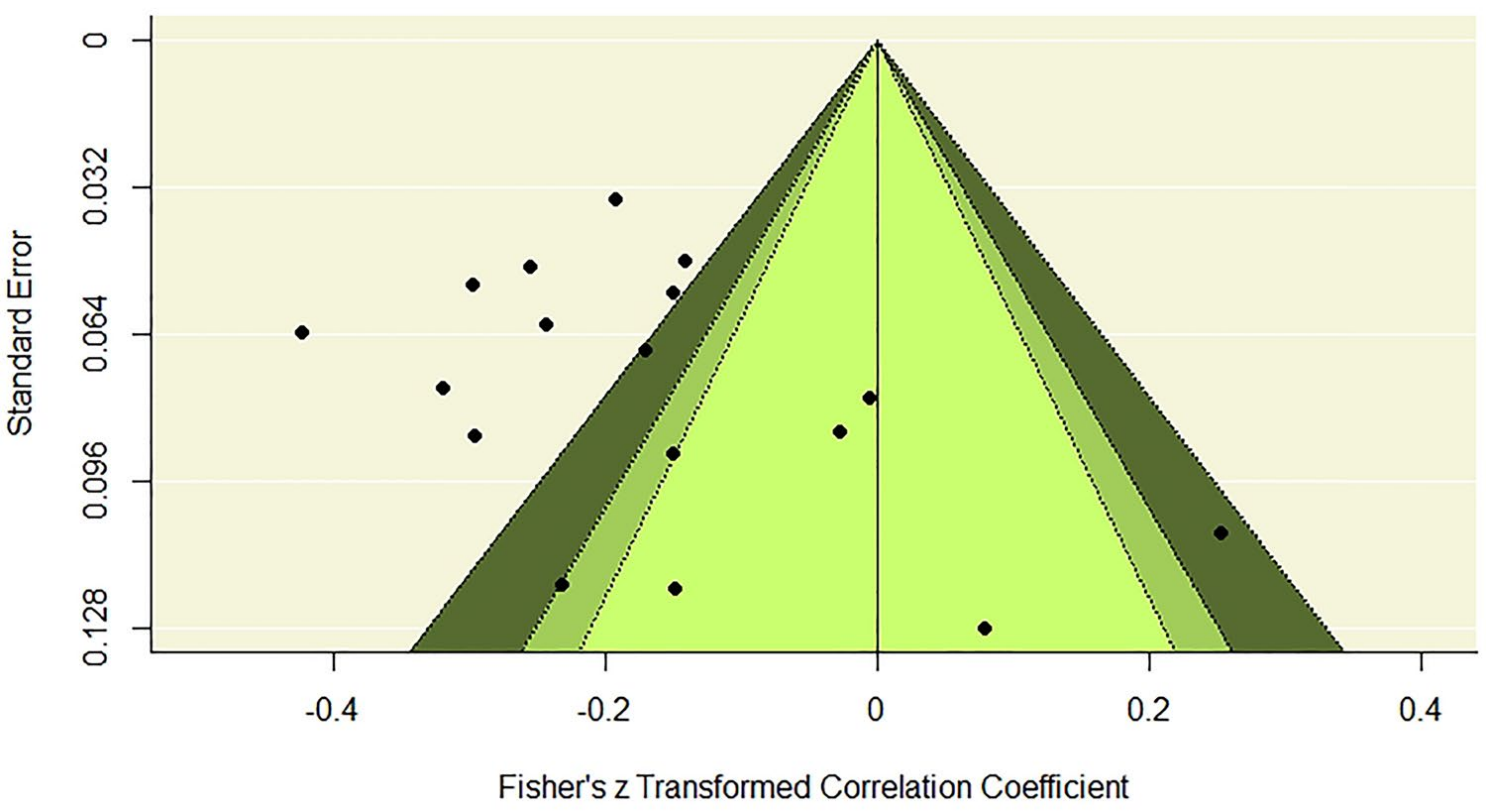
Heterogeneity diagnostics using a contour-enhanced funnel plot. The effects in the white zone are greater than *p* = 0.10; the effects in the adjacent light gray zone are between *p* = 0.10 and *p* = 0.05; the effects in the darker gray zone are between *p* = 0.05 and *p* = 0.01; the effects outside this zone, marked with the light gray, are smaller than *p* = 0.01

We examined the potential publication bias effect with a contour-enhanced funnel plot. Figure 4 presents the results, which show that positive relationships between negative indicators of well-being and PTG are present only in studies with small sample sizes. Otherwise, stronger and weaker negative relationships were present in studies with smaller and larger samples. We concluded that no publication bias adjustment was necessary.

### Effect sizes and heterogeneity

**Fig. 5:**
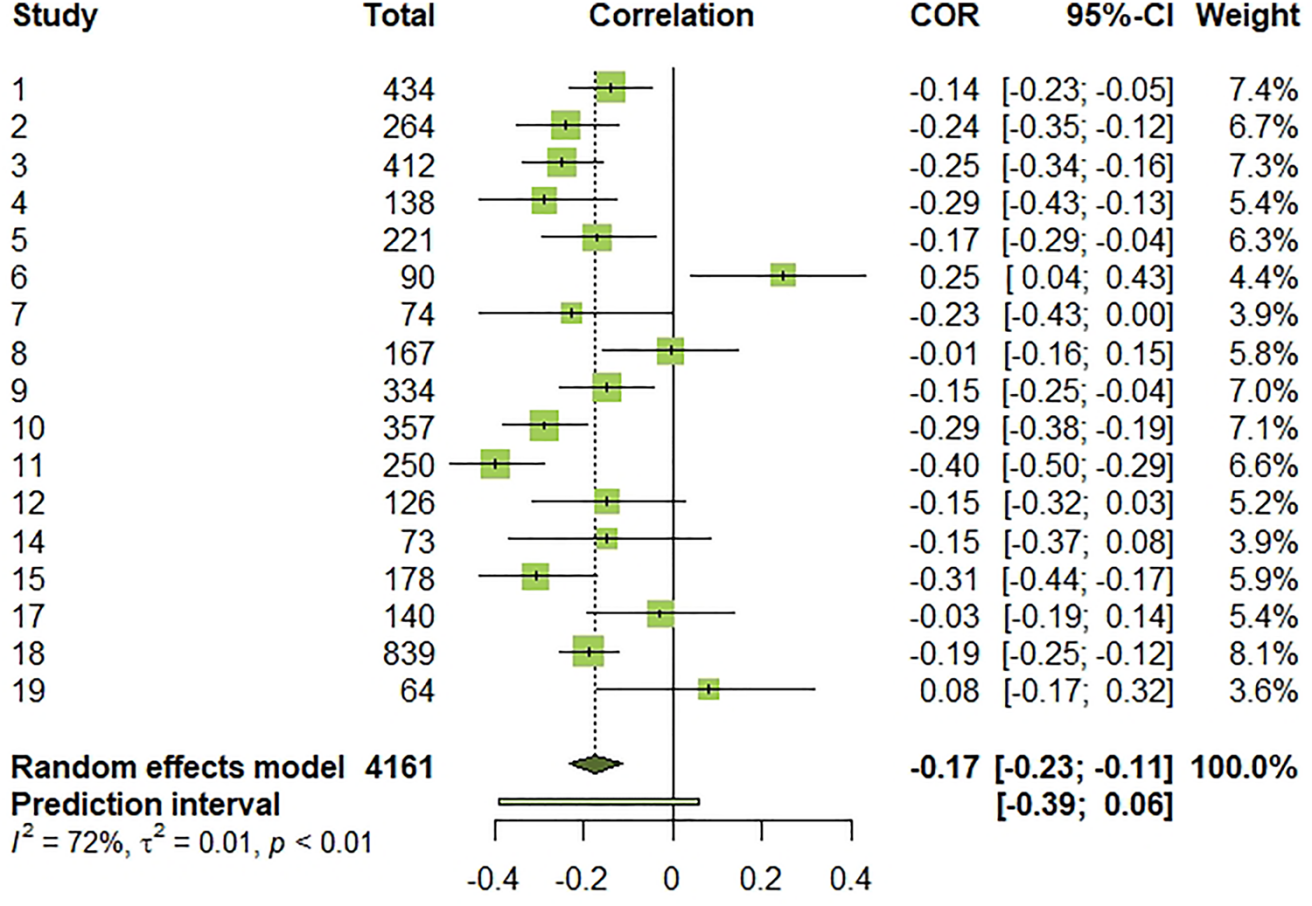
Forest plot of effect sizes for individual studies, overall estimated effect, and 95% prediction interval. τ^2^: between-study variance. I^2^: I-squared statistic of heterogeneity

The effect sizes for individual studies ranged from − 0.40 to 0.25. Heterogeneity was statistically significant, (*Q*(16) = 56.93, *p* < 0.001, *I*^2^ = 71.9% [54.2%; 82.7%]), indicating that 72% of the total variation in estimated effects was due to between-study variation, which was close to being high [80]. The random-effects pooled estimate revealed a negative and weak-size association [81] between PTG and negative subjective well-being (*r* = − 0.18, 95% CI [− 0.23; − 0.11]). However, a 95% prediction interval [− 0.41; 0.06] informing the range of true effects in similar future studies suggests that this association may be from negative to null [82]. The forest plot below summarizes effect sizes for individual studies and meta-analysis (Fig. 5).

### Moderators

In the next step, possible moderators of the obtained effect size were examined through meta-regression. They included publication year and socio-demographic and clinical characteristics. There was no evidence of variation in effect size due to publication year (*B* = 0.01, *p* > 0.05). The observed effect size also did not change with percentage of male participants in the study (*B* = − 0.01, *p* > 0.05), participants’ mean age (*B* = 0.01, *p* > 0.05), being in a stable relationship (*B* = − 0.17, *p* > 0.05), higher education (*B* = 0.28, *p* > 0.05), or stable employment (*B* = 0.03, *p* > 0.05). Similarly, statistically insignificant results were noted for the percentage of participants with heterosexual orientation (*B* = 0.22, *p* > 0.05) and Caucasian ethnicity versus other ethnicities (*B* = − 0.06, *p* > 0.05). For clinical variables, all the effects were insignificant, including CD4 (*B* = − 0.01, *p* > 0.05), mean viral load (*B* = 0.11, *p* > 0.05), mean time since diagnosis (*B* = − 0.01, *p* > 0.05), and AIDS status (*B* = − 0.08, *p* > 0.05). Thus, no moderators were identified.

## Meta-analysis: PTG and positive HIV-related well-being indicators

**Fig. 6:**
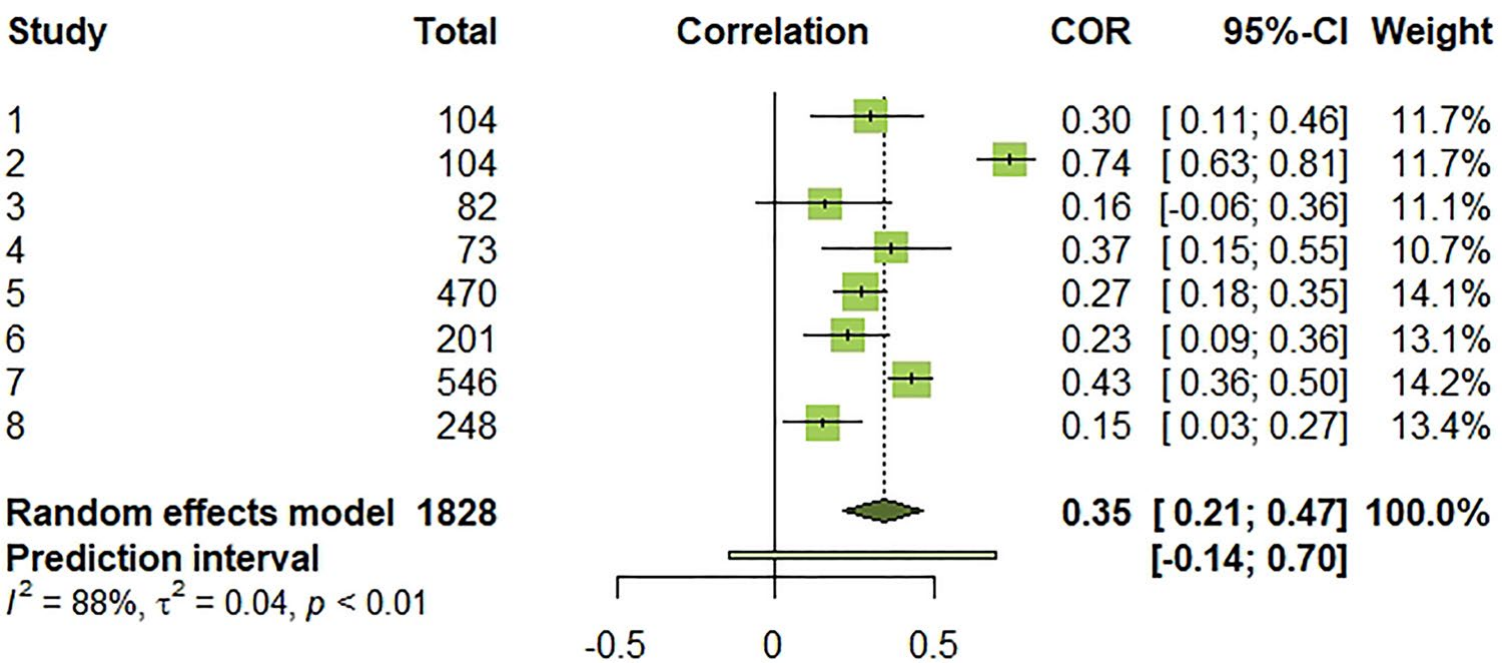
Forest plot of effect sizes for individual studies, overall estimated effect, and 95% prediction interval. τ^2^: between-study variance. I^2^: I-squared statistic of heterogeneity

Second, we conducted a meta-analysis for the studies based on positive indicators of well-being (*k* = 8). The effect sizes for individual studies ranged from 0.15 to 0.74. Heterogeneity was also statistically significant (*Q*(7) = 60.14, *p* < 0.001, *I*^2^ = 88.4% [79.4%; 93.4%]), indicating that 88% of the total variation in estimated effects was due to between-study variation, which is high [80]. The random-effects pooled estimate revealed a positive and medium-size association [81] between PTG and positive aspects of PLWH’s well-being (*r* = 0.35, 95% CI [0.21; 0.47]). However, a 95% prediction interval [− 0.14; 0.70] informing the range of true effects in similar future studies suggests that this association may be negative to positive, including null [82]. The forest plot below summarizes effect sizes for individual studies and for meta-analysis (Fig. 6).

## Summary of main findings

The systematic review and meta-analysis objective was to synthesize, analyze, and critically review existing studies on the relationship between posttraumatic growth and psychological well-being among PLWH. After the selection process, we included 27 articles published between 2004 and 2021 in the analysis. Selected papers met the selection criteria concerning the content and quality of the studies. Two studies significantly deviated from all other studies and, on this basis, were excluded from further analysis. The meta-analysis investigated reported effect sizes. For 19 studies implementing negative well-being indicators, a meta-analysis revealed a negative weak-size association between PTG and the indicators (*r* = − 18,95% CI [− 0.23; − 0.11]). We detected positive medium-size associations between PTG and positive well-being indicators for eight studies that focused on positive well-being indicators (*r* = 0.36, 95% CI [0.21; 0.47]). Specifically, the relationship between PTG and positive well-being indicators was more substantial than the link between PTG and negative well-being measures in these patients. We identified no variables as moderators of the studied relationships. Moreover, there was no evidence of variation in effect size due to publication year (*B* = 0.01, *p* > 0.05), which implies a stable relationship between PTG and well-being despite the advancement of medical treatment [14, 32].

The systematic review of 27 articles provided some evidence of the role of socio-demographic and medical variables in the relationship between PTG and PLWH’s well-being. While 14 studies included in the review identified no significant covariates, in 13 studies, some socio-demographic or medical variables mattered for the studied PTG-well-being association among PLWH.

First, there were significant relationships between PTG and socio-demographic data, such as gender, ethnicity, age, and sexual orientation. Four studies identified higher PTG levels in women [27, 55, 59, 63], whereas one study reported higher PTG levels in men [75]. The inconsistency of the results may be due to cultural differences, as lower PTG levels are present in a sample of African women. We mainly examined PTG among PLWH in homogenous Western cultural groups, and the interaction between PTG and gender needs further investigation in other cultural contexts [14]. Thereby, we observed significant differences in PTG levels across different ethnic groups. Three studies reported significantly lower PTG levels in Caucasians compared to other ethnic groups. One study revealed the moderating role of Caucasian ethnicity in the association between benefit finding and depression [66]. Indicated groups with the most elevated PTG levels differed across selected studies. One revealed the highest PTG levels in Hispanics [54] and two—highest PTG levels in African Americans [63, 59]. Three studies identified both positive [57, 78] and negative [72] associations between PTG level and age. In the previous study, the sample consisted exclusively of homosexual and bisexual participants. Belonging to sexual minorities could explain the negative association between age and PTG level, as elevated stigma and worse well-being are observed predominantly in this group of PLWH (e.g., [83]. However, only one of the eligible studies included in the systematic review identified sexual orientation as a significant covariate of well-being in PLWH. Kamen et al. [63] reported higher stigma levels in heterosexuals. This result is in line with some studies (e.g., [84] and can also be explained by a stigma accumulation in ethnic minority groups. Only one study by Rzeszutek et al. [74] showed an interesting moderating role of relationship status for the association between life satisfaction and PTG. The positive link between the two variables was significant only in single participants. In most studies, an intimate relationship is the most important source of social support for PLWH and is associated with positive well-being indicators [85]. However, it is in line with the theoretical PTG framework that more vulnerable populations may experience the phenomenon [1, 6].

The last group of socio-demographic covariates, i.e., education and employment, showed relatively homogenous effects on well-being. Three studies revealed significant associations between higher education and PTG. Two reported a negative association between the two variables [75, 65], and another indicated a stronger association between SWLS and PTG in less educated participants [68]. Some studies show possible adverse effects of higher education on PLWH’s well-being, as education level may be associated with more intense perceived stigmatization (e.g., [86]).

Clinical variables such as CD4 mean, detectable viral load, years since diagnosis or treatment years, and AIDS status were the last group of analyzed covariates. However, one should bear in mind two issues. First, they should also be treated with caution, as they were self-reported in most studies. Second, usually, they were unrelated to PTG among study participants, or ethnicity moderated the relationship with PTG (e.g., [55]. In particular, we did not find any evidence on the moderating role of time since HIV diagnosis on PTG in this patient group, which the previous review on that issue suggested [14]. It seems that PTG in the context of life-threatening illnesses like HIV/AIDS is not strictly associated with the progression of the disease itself, but rather socio-demographic or psychosocial variables, which was also shown in the case of cancer-related PTG [13].

## Limitations and future directions

The systematic review and meta-analysis resulted from an exhaustive literature search and study selection that followed the PRISMA selection criteria. However, the results of our work are not free of limitations. For example, we have excluded studies published in languages other than English. We have also excluded qualitative literature that may hinder our understanding of underlying mechanisms linking PTG and well-being in PLWH. Further significant limitations of our work are associated with operationalizations of HIV-related PTG, well-being, or distress. First, we observe inconsistency of PTG models across included studies. Although most eligible studies followed the PTG model by Tedeschi and Calhoun [1, 6], and the majority of studies had PTG as an outcome variable in their models, some treated PTG as a predictor variable and included well-being or distress outcome variables. Second, we found different tools with different psychometric characteristics to measure PTG and well-being. In particular, we noticed various growth measurement tools (e.g., PTG, benefit finding etc.), which reflects still existing conceptual heterogeneity or even theoretical chaos associated with operationalization of the term *growth after trauma* in the literature [10]. Third, relevant statistical details for the meta-analysis were, in most cases, available only for global PTG scores as well as for the studied HIV-related well-being and distress issues, despite the assessment of subscales. In other words, our review and meta-analysis indicate, once again, the problem of the lack of a conclusive theoretically and empirically validated model and measurement of PTG in general [8, 10].

In the context of life-threatening illnesses such as HIV/AIDS, authors underlined the importance of clarifying the associations between PTG and both physical and psychological benefits [4, 14, 33]. To this day, research in the PTG has been highly inconclusive and reporting positive [43], negative [3, 87], or no relationship between PTG and distress indicators [88]. Despite the variability study results mentioned above, the meta-analysis showed a medium-size positive relationship between PTG and positive well-being indicators and a small-size negative relationship between PTG and negative indicators of well-being among PWLH. This is in line with a hypothesis that PTG is associated with positive adjustment in time [14]. However, the available data cannot explain the mechanisms behind PTG processes. Also, our systematic review confirmed the role of sample characteristics such as age, gender, ethnicity, and medical indicators. Nevertheless, the effects of significant covariates on PTG level were heterogeneous. Consequently, future research should focus on how these individual differences relate to PTG and adjustment, as well as their role in the PTG process [14, 33].

Future research should implement new directions to clarify the role of PTG in PLWH. As PTG is a process of change, longitudinal studies are crucial for explaining PTG mechanisms. Also, future research should include mediators and moderators and test beyond linear relationships. Finally, future studies on PTG in PLWH can take advantage of the newest PTG research recommendations that address theoretical and methodological aspects of PTG research [10]. PTG studies should specify the types of life events being examined in a study (e.g., receiving an HIV diagnosis, entering the AIDS stage). They should investigate the influence of adversity on traits sensitive to change, explore the relation between adversity and narrative identity, and develop theories (and their measures) sensitive to cultural context [10].

## Conclusions

Our meta-analysis and systematic review indicate that PTG is a worthy phenomenon and promising area of research that therapists can implement in their clinical practice [14]. However, PTG research among PLWH shares the same shortcomings as the overall PTG literature [8], with the additional complication of the ongoing nature of illness-related trauma and related measurement difficulties [13]. Thus, one should replace retrospective, self-reported PTG measures, which almost exclusively dominate our review and meta-analysis by prospective study design, ideally accompanied by the ecological momentary assessment models to understand PTG dynamics in time [10]. PTG research should also consider new theoretical PTG conceptualizations, such as the measurement of posttraumatic growth (PTG) and posttraumatic depreciation (PTD). PTD is the parallel negative reflection of PTG and is defined as negative changes in the same PTG domains among people exposed to traumatic events [89]. Recent authors found the independence of these two constructs, which could also have different correlates and lead to opposite well-being outcomes [90]. Researchers should treat these as parallel but independent experiences after trauma, which are uniquely related to the well-being of trauma survivors [91].

From a clinical perspective, our systematic review and meta-analysis revealed the complexity of growth process in the case of struggling with HIV infection, but also showed the importance of PTG in enhancing PLWH’s well-being. Recently, some authors created PTG promotion interventions in the case of struggling with life-threatening illness [92, 93]. Psychological interventions to enhance PTG among PLWH need to consider vast social context of living HIV related with stigma, which remains dynamic since the beginning of the pandemic 40 years ago [94]. Also, in the case of PLWH, the promotion PTG is associated with specific goals, including not only enhancing compliance with the rigors of treatment, but also learning to benefit from close relationships [33]. Finally, the access to psychological care for these patients is still very limited and acknowledgement of its meaning for PLWH is still a challenge in the medical care for this patient group [31].
